# Adherence to the Singapore Integrated 24 h Activity Guidelines for Pre-Primary School Children Before, During and After the COVID-19 Lockdown in Singapore

**DOI:** 10.3390/sports13020032

**Published:** 2025-01-23

**Authors:** Seow Ting Low, Terence Buan Kiong Chua, Dan Li, Michael Chia

**Affiliations:** 1 Physical Education & Sports Science, National Institute of Education, Nanyang Technological University, Singapore 637616, Singapore; nie22.lst@e.ntu.edu.sg (S.T.L.); terencechuabk@gmail.com (T.B.K.C.); 2 School of Physical Education, Hunan Normal University, Changsha 410012, China; lidan97@hunnu.edu.cn

**Keywords:** physical activity, sleep, screen time, 24 h activity guidelines, COVID-19 pandemic, pre-primary school children

## Abstract

The COVID-19 pandemic has significantly disrupted the lives of pre-primary school children in Singapore where increased infection rates prompted lockdown measures that altered children’s daily routines. This study aimed to evaluate the impact of the pandemic on the lifestyle behaviours and health quality of 3134 children aged 5 to 6 years across three periods: pre-COVID, COVID-19 lockdown, and COVID-19 endemicity. Data were collected using the Surveillance of Digital Media Habits in Early Childhood Questionnaire (SMALLQ^®^) to measure on- and off-screen media habits of children and the Pediatric Quality of Life Inventory (PaedQL) to assess children’s health quality. Adherence to physical activity (PA) guidelines dropped from 32.7% pre-COVID to 27.4% during lockdown but improved to 34.4% in endemicity (*p* < 0.05). Sleep (SL) adherence followed a similar pattern, decreasing from 33.4% to 27.9% before rising to 40.6% (*p* < 0.05). Screen time (ST) adherence significantly declined during lockdown (16.7% to 10.8%, *p* < 0.001). Weak positive correlations with all PaedQL metrics were observed across periods, except during endemicity (*p* < 0.05). Concerted efforts involving key stakeholders must be made to mitigate the negative effects of the pandemic on children’s lifestyle behaviours and QoL, ensuring they are better prepared for the transition to primary school.

## 1. Introduction

### 1.1. Pre-Primary School Children and Quality of Life

Early-life habits play a crucial role in shaping long-term health outcomes, both positively and negatively [1,2]. The concept of sensitive periods for development and transition highlights that pre-primary years are a critical phase of heightened receptivity for cognitive, social, emotional, and physical development [3]. During this time, children are especially skilled at acquiring new knowledge and abilities, making it vital for educators and caregivers to provide enriching experiences to build the right attitude toward a good quality of life [4]. Positive experiences, such as proper nutrition, emotional support, and stable environments, promote healthy development and future well-being [5,6,7]. In contrast, adverse experiences like neglect can have lasting negative effects [4]. Therefore, it is crucial to cultivate healthy habits and positive lifestyle behaviours during the early years. Early support sets the foundation for long-term well-being, helping children develop the skills and resilience needed to maintain good health [3,8].

The transition from preschool to primary school education is a crucial period in a child’s growth and development [9], marking a significant shift in their learning environment and social interactions [10,11]. This transition encompasses a range of emotional, cognitive, and social adjustments that can significantly impact a child’s educational trajectory. Research indicates that a well-structured transition can enhance children’s readiness for school, thereby facilitating their academic success and overall well-being [12,13].

Crucial to a successful transition is also the quality of life (QoL). Emotional and behavioural aspects of QoL and physical health are crucial for pre-primary school children transitioning to school [14,15]. Social skills also play a crucial role in the transition to school, as they are essential for forming relationships with peers and teachers [16,17]. The development of social skills during preschool is linked to children’s readiness for school, as children who can communicate effectively and interact positively with others are better prepared to navigate the social dynamics of a classroom [18].

Moreover, the physical health of pre-primary school children is integral to their QoL and school readiness. Adequate nutrition, physical activity (PA), and sleep (SL) are essential for optimal development and functioning [5,6,19]. Research has demonstrated that children who engage in regular PA and maintain a balanced diet exhibit better cognitive and emotional outcomes, which are crucial for successful school transitions [20,21]. Conversely, children facing health challenges may experience difficulties in adjusting to school, underscoring the importance of addressing health-related QoL factors during pre-primary school years [22].

Therefore, adherence to 24 h activity guidelines, which encompass PA, sedentary behaviour, and SL, can influence the QoL of young children. These guidelines promote a balanced lifestyle that integrates sufficient SL, regular PA, and limited sedentary time, which collectively contributes to improved health outcomes and overall well-being [23,24], all of which can enhance children’s ability to adapt to new educational challenges, ultimately contributing to their overall QoL and academic success. Therefore, it is crucial to encourage adherence to these guidelines, especially in early childhood, as adherence often decreases when children begin primary school due to the increased demands of formal education [12,13].

### 1.2. The COVID-19 Pandemic

However, unforeseen circumstances can sometimes arise, disrupting children’s ability to adhere to the 24 h activity guidelines. A prominent example is in 2020 when the world was shaken by the emergence of a novel disease known as coronavirus disease 2019 (COVID-19). On 30 January, the World Health Organization (WHO) declared COVID-19 a global health emergency, and by 11 March 2021, it had escalated to a pandemic [25]. The emergence of the COVID-19 pandemic has profoundly affected lifestyle behaviours worldwide, including those of pre-primary school children.

Around the world, many countries adopted various lockdown restrictions to curb the spread of the virus. As a result, public facilities, workplaces and educational facilities were either closed or operated with restricted hours, affecting all, including pre-primary children [26,27,28,29,30].

Studies have shown changes in various aspects of children’s lives, including SL patterns, PA, screen time (ST), and mental health before, during, and after the pandemic [27,28,31,32,33]. The closure of schools and kindergartens during the pandemic led to alterations in children’s daily routines, such as increased sedentary behaviour and irregular SL schedules [34,35,36].

In Japan, PA among preschool children dropped notably, with weekday and weekend playtime declining by 8.5% and 15.3%, respectively, following the onset of the pandemic [27]. Similarly, China experienced a decrease in adherence to PA guidelines, from 35.7% to 22.5% [37]. ST adherence among Japanese children also fell from 27.2% before the pandemic to 19.9% during it [27]. In China, ST usage was found to have increased from 47.5% to 85.7% during the pandemic [37]. A study in Indonesia also revealed a staggering 150.0% increase in average daily screen time during the COVID-19 pandemic [38].

These changes have also been linked to negative effects on mental health, with children expressing worries and experiencing increased stress levels [39,40,41]. The prolonged home confinement led to disruptions in established routines, which are crucial for young children’s emotional and psychological well-being [27,33]. Furthermore, the pandemic has been associated with an increased incidence of overweight and obesity among pre-primary school children, possibly exacerbated by disrupted daily structures, mealtimes, and PA due to school closures [42,43,44]. In China, significant weight and height changes were observed among pre-primary children during school closures, indicating a worrying trend towards obesity [45]. Similarly, in Sweden, a marked increase in the incidence of overweight and obese children was found due to reduced PA and altered dietary habits during the pandemic [43].

Among 5- and 6-year-old pre-primary children, the impact of the COVID-19 pandemic on children’s exposome profile, encompassing factors like PA and ST, has been evident, highlighting the broad-reaching effects of the pandemic on children’s overall well-being [46].

### 1.3. Singapore Context

In Singapore, the surge in COVID-19 cases also led the Singapore government to enforce preventive lockdown measures, causing major disruptions to children’s daily routines.

With the rise in the number of COVID-19 cases in 2020, Singapore introduced a lockdown, known as the circuit breaker, to curb the spread of COVID-19. During the period between April 2020 to June 2020, workplaces shifted to remote operations, food establishments were restricted to take-away services, and schools, including preschools, moved to home-based learning [47]. Over the next two years, from 2020 to 2021, Singapore periodically introduced and lifted various restrictions, such as banning dining in, enforcing remote work and learning, and limiting gathering sizes in response to case surges. These measures were applied from June to December 2020, then again from May to June 2021, and from July to August 2021. By March 2022, outdoor mask requirements were lifted and restrictions were eased as we moved into the endemicity of COVID-19 [47].

### 1.4. Singapore Integrated 24 h Activity Guidelines for Early Childhood

In 2022, Singapore introduced the Singapore Integrated 24 h Activity Guidelines for Early Childhood [48]. Unlike the World Health Organization (WHO) 24 h Movement Guidelines, which categorise children and adolescents aged 5 to 17 under the same age group with uniform recommendations [49]. Singapore’s guidelines are more specifically designed for different age ranges: infants (under 1 year), toddlers (1 to under 3 years), and preschoolers (3 to under 7 years). These guidelines also include dietary recommendations, which will not be evaluated in the current study as they were recently introduced.

For children aged 5 to 6, the WHO recommends at least 60 min of moderate-to-vigorous PA daily, with muscle and bone-strengthening activities at least three times a week [49]. While the WHO suggests limiting sedentary ST, it does not specify a daily limit. Additionally, children are advised to get 9 to 11 h of uninterrupted SL each night.

In succinct terms, the Singapore Integrated 24 h Activity Guidelines for 5- to 6-year-old pre-primary school children are the following:

For pre-schoolers (3 to <7 years):
Physical Activity:
  - Accumulate a minimum of 180 min of physical activity throughout the day, in a safe environment.
  - At least 60 min should be of moderate to vigorous intensity; more is beneficial.
Sleep:
  - Ensure 10 to 13 h of sleep per day for children aged 3 to 5 years old, or 9 to 11 h for 6-year-olds.
Screen Time:
  - Limit recreational screen time on any device to less than 1 h.

### 1.5. Purpose and Research Agenda

The pre-primary school period, typically for children aged 5 to 6 years is a critical stage of development that lays the foundation for future academic and social success. At this period, children undergo significant cognitive, emotional, and social growth, which prepares them for the more structured and demanding environment of formal schooling. Given the importance of building the right lifestyle behaviours to promote healthy development of the body and mind, it is essential to encourage greater adherence to meeting the recommended 24 h activity guidelines.

Singapore introduced its own 24 h activity guidelines for early childhood in 2021 to address the nation’s specific health challenges (e.g., rapidly ageing population), cultural contexts (e.g., multi-racial) and lifestyle habits (e.g., high rates of diabetes in middle age groups). The development of these guidelines was prompted by rising concerns about childhood obesity, increasing sedentary behaviour and a lack of PA among young children. The initiative aimed to promote a healthier balance of PA, SL and ST to support the holistic well-being and development of preschool-aged children.

However, the COVID-19 pandemic further exacerbated these issues, disrupting children’s routines and negatively impacting their ability to meet recommended levels of PA, SL, and ST [50]. The ongoing effects of these disruptions highlight the critical need to reassess children’s adherence to these guidelines, in order to develop strategies that can mitigate the pandemic’s impact on their health and well-being.

Examining Singapore as a case study during the COVID-19 pandemic provides unique insights into the multifaceted impacts of the pandemic on pre-primary school children. With its robust public health infrastructure and proactive government response during the pandemic [51], Singapore created a distinct environment to analyse how the pandemic has affected children’s lifestyles and health quality. The country’s swift response, such as early lockdowns, widespread testing, and contact tracing, as well as the implementation of various support initiatives aimed at supporting families and children during the pandemic, creates a unique setting for observing changes in children’s behaviours and its effects on health outcomes [52]. Lessons learnt from Singapore’s experience with the COVID-19 pandemic can also inform future strategies, and these insights can guide future policies that protect the overall well-being of children in the face of future global health crises.

Therefore, this research sought to evaluate how the COVID-19 pandemic has affected Singapore’s pre-primary school children’s lifestyle behaviours at three critical stages: before, during, and after the COVID-19 pandemic lockdown. By comparing these periods, this research aimed to provide a comprehensive understanding of the changes in children’s PA, SL patterns, and ST, and how these behaviours are associated with their QoL. This information will help identify potential areas for intervention and opportunities for lifestyle amelioration in the daily lives of pre-primary school children in the COVID-19 endemic period.

### 1.6. Research Questions

1. To what extent did the COVID-19 pandemic affect pre-primary children’s adherence to the Singapore Integrated 24 h Activity Guidelines for (a) physical activity, (b) sedentary behaviour, (c) sleep and (d) screen time?
2. What is the association between the number of activity guidelines met and the corresponding quality of life of pre-primary school children?

## 2. Materials and Methods

### 2.1. Study Design

This was a cross-sectional study designed to assess the impact of COVID-19 pandemic on pre-primary school children in Singapore over 5 years across three time periods, including the pre-pandemic period of 2018–2019, the pandemic lockdown period of 2020–2021 and the COVID-19 endemic period of 2022, where data were collected. In the context of the research, the pre-COVID period represented life before COVID-19 emerged. The COVID-19 pandemic lockdown started in the year the COVID-19 pandemic was declared a global pandemic, leading to widespread lockdowns and restrictions to curb the spread of the virus. In the COVID-19 endemic period, vaccines became widely available, and more people acquired immunity, COVID-19 continued to circulate in communities but at more manageable levels, most restrictions were lifted, and people adjusted to a new phase of coexisting with the virus.

In the research design, parent-reported changes to pre-primary school children’s adherence to PA levels—indoor and outdoor playtime, moderate-to-vigorous intensity activity, SL patterns and ST usage over the sign-posted periods of pre-COVID-19, COVID-19 pandemic and COVID-19 endemicity were monitored. In the same periods, parent-reported health-related QoL of pre-primary school children was also collected.

#### 2.1.1. Research Ethical Clearance and Participants

Institutional ethical clearance for the research was granted (IRB-2017-09-036).

Prior to the commencement of the study, parent participants of children from these preschools who enrolled on the study gave informed consent to voluntary participation. Throughout the period of the research, the researchers abided by the Singapore Personal Data Protection Act (2012) and ensured anonymity of data from all participants.

Recruitment took place in collaboration with the leadership team of the preschool operators, who helped distribute informed consent forms to parents or caregivers of eligible children. Participants were parents of pre-primary school children aged 5 to 6 years. They were recruited from seven preschool operators, five of which were preschool anchor operators offering subsidised and high-quality early childhood education, particularly targeting lower-income and disadvantaged families. The remaining two preschools were operated by private enterprises that had smaller student enrolments.

Data were collected before, during and after the COVID-19 pandemic. The questionnaires used in the study were hosted on Qualtrics, an online secure platform, and the link to the survey was shared with the parents through the school’s administrative teams. The online survey was conducted anonymously, ensuring that no personally identifiable information such as names, addresses, phone numbers, or email addresses was collected. Parents were only asked to provide basic demographic information necessary for the study (e.g., age, gender, and preschool), which was aggregated and anonymised in the analysis.

#### 2.1.2. Questionnaires

##### Surveillance of digital Media hAbits in earLy childhood Questionnaire (SMALLQ^®^)

The SMALLQ^®^ was hosted online on a secure platform, Qualtrics, that was approved by the university. Parents of pre-primary school-aged children were contacted and invited to participate in the online survey via letters with a QR code to complete the survey, sent through the participating preschool operators in Singapore.

The SMALLQ^®^ is a 25-item questionnaire designed to gather data from parents about their children’s digital media habits (screen time, type, and purpose) and non-digital media activities (indoor and outdoor play, SL duration, and quality) on both weekdays and weekends, based on a 7-day recall [53]. It consists of three main sections: (a) digital media use by both parent and child; (b) off-screen activities of the child; and (c) demographic information of the parent and child. The reliability and internal consistency of SMALLQ^®^ have been established. The Cronbach’s alpha values, based on parent-reported child habits, were 0.78 for PA, 0.74 for digital media use, ST, and 0.69 for SL on both weekdays and weekends [53]. Overall, the SMALLQ^®^ had sufficient face and content validity and acceptable internal consistency for assessing lifestyle behaviours in pre-primary school children.

*Demographic Variables*

SMALLQ^®^ was used to survey the participants’ demographical variables. Information on the child’s sex, age and ethnicity was obtained from their parents.

*Screen* *Time*

Parents were asked to provide details of their child’s screen media on a typical weekday and weekend day. This included the duration and the purpose of screen media use (e.g., education, entertainment, media creation, communication) on fixed screen and mobile screens.

*Indoor and Outdoor Play Time*

Parents reported on their child’s PA on a typical weekday and weekend day. This was split into indoor play as well as outdoor play. Additionally, parents were asked to estimate the percentage of time spent on indoor and outdoor play that caused their child to breathe harder and faster.

*Sleep Duration*

Parents were asked to report on the average duration of their child’s night-time SL on a typical weekday and weekend day.

##### Paediatrics Quality of Life Inventory (PedsQL™)

The acute 7-day recall version of the PedsQL™ was used to assess parent-reported health-related QoL of children [54]. The brief 23-item measure yielded two summary scores—the psychosocial health summary score and physical health summary score—and a total scale score. These scales sought input from parents on five scales: (1) Physical Functioning (8 items), (2) Emotional Functioning (5 items), (3) Social Functioning (5 items) and (4) School Functioning (5 items).

Each subscale was based on a 5-point Likert scale ranging from “never” to “almost always a problem”, with scores of 0, 25, 50, 75, and 100 assigned accordingly. Each scale score was then computed by dividing the sum of item scores by the number of items. The total health score, ranging from 0 to 100, combined Psychosocial Health and Physical Health scores, with higher scores indicating better QoL. The PedsQL 4.0 has been validated extensively in various populations, demonstrating reliability and cross-cultural applicability and has been found to have an internal consistency reliability of 0.90 for the Parent Proxy Report [54].

### 2.2. Statistical Analyses

All statistical analyses were conducted using JASP Team (2024). JASP (Version 0.19.0) [Computer software] and the significance level was set at *p* < 0.05.

Data with more than 90% missing values were excluded from analysis. Outliers were present—some values were extreme but not impossible values (e.g., more than 24 h a day) and were therefore kept in the analysis. A total of 3134 complete and valid responses were received (*n* = 1585 from pre-COVID, *n* = 1209 from COVID-19 lockdown and *n* = 340 from COVID-19 endemicity).

Descriptive analyses were performed to outline the demographic composition of pre-primary school children across the three time periods and to compute the mean duration of physical play, screen media use, and SL per day, using a 1:1 ratio for weekdays and weekends.

Data normality was assessed using the Shapiro–Wilk test, which indicated that not all variables followed a normal distribution. Given that the variables on PA, ST and SL did not follow a normal distribution and exhibited skewed distributions, the Kruskal–Wallis H-test with Dunn’s post hoc test was utilised to compare the differences in the PA, ST, and SL duration across the three time periods, without relying on parametric assumptions. Using this approach ensured a more robust and reliable result.

Chi-square test was used to compare the proportion of children who met each guideline—PA, SL, and ST—across the three time periods as well as to determine if there were any significant differences in the proportion of children meeting guidelines across the three time periods. These categories included children meeting none, one, two, or all three of the recommended guidelines.

Additionally, the correlation between QoL scores and number of guidelines met (none vs. one vs. two vs. three) was examined using partial correlations while controlling for child’s age and sex. These analyses aimed to investigate the impact of adherence to the Singapore Integrated 24 h Activity Guidelines on both guideline adherence and QoL among children.

## 3. Results

Table 1 provides the demographic characteristics of pre-primary school children across the three time periods of COVID-19. Among the 3134 participants, 1517 were boys, 1599 were girls, and 18 did not specify their gender. Ethnically, the largest group was Chinese (*n* = 1907), followed by Malays (*n* = 547), Indians (*n* = 418), Others (*n* = 245) and Eurasians (*n* = 8), with nine not specifying their ethnicity. The most common age group was 5-year-old participants (*n* = 2117), compared to 6-year-olds (*n* = 1453). Table 1 provides a summary of the demographic distribution of the study’s participants across the three time periods.

Table 2 presents the mean and standard deviation (SD) for PA, SL, and ST of children on an average day across the three time periods of COVID-19.

PA, including indoor, outdoor, and energetic play, decreased significantly during the COVID-19 lockdown compared to pre-COVID-19 and endemic periods (*p* < 0.01). SL duration showed a slight increase in the endemic period compared to the lockdown (*p* < 0.05). ST increased during the lockdown (*p* < 0.05) but slightly declined in the endemic phase, with no significant difference between the two. These results highlight the temporary impact of the lockdown on children’s activity patterns, with partial recovery post-lockdown.

Table 3 provides an overview of the percentage of children meeting the Singapore Integrated 24 h Activity Guidelines for PA, SL, and ST across the three time periods.

The percentage of children meeting PA guidelines decreased during the COVID-19 lockdown compared to pre-COVID-19 and endemic periods (*p* < 0.01). Adherence to SL guidelines improved over time, with the highest proportion observed during the endemic phase (*p* < 0.01). However, adherence to ST guidelines dropped significantly during the lockdown and remained low in the endemic phase compared to pre-COVID-19 levels (*p* < 0.001). These findings reflect the varying impacts of the COVID-19 pandemic on children’s health behaviours.

**Figure 1:**
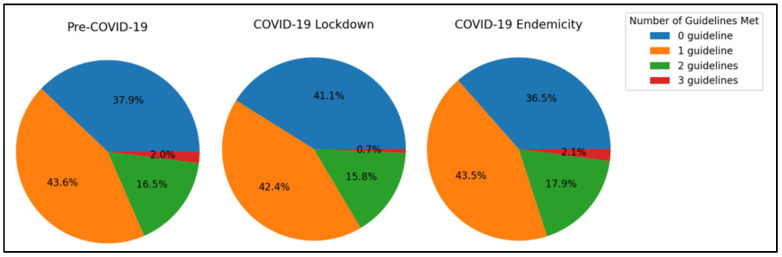
Percentage of children who met none, one, two or all three guidelines each time period.

Table 4 and Figure 1 show the number of children who met none, one, two, or all three guidelines for PA, SL, and ST across the three time periods.

A chi-square test (*p* = 0.0482) confirms the differences in guideline adherence are statistically significant.

During the COVID-19 lockdown, there was an increase in the proportion of children meeting no guidelines, while adherence to one guideline remained stable across all periods. The percentage of children meeting two guidelines showed little change, but the proportion meeting all three guidelines dropped significantly during the lockdown. This decline was observed to a lesser extent in the endemic phase, indicating a partial recovery in guideline adherence after the lockdown.

**Figure 2:**
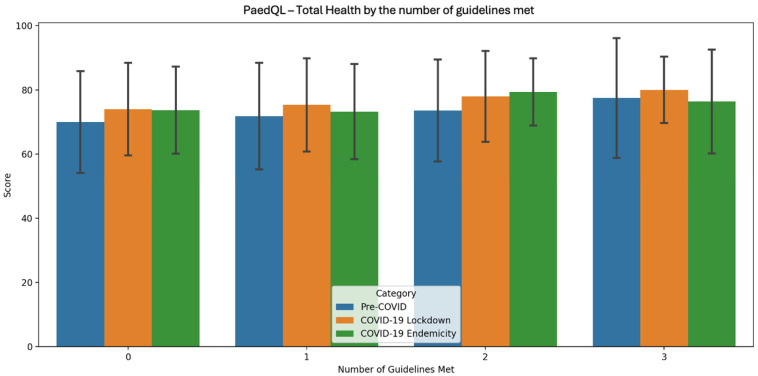
PaedQL score for total health by the number of guidelines met across each time period of COVID-19.

**Figure 3:**
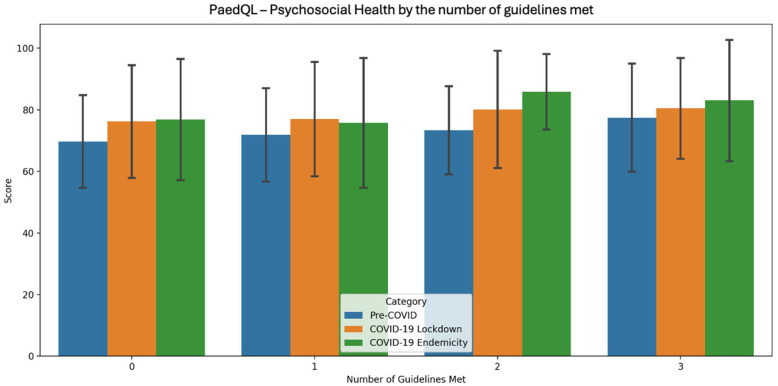
PaedQL score for psychosocial health by the number of guidelines met across each time period of COVID-19.

**Figure 4:**
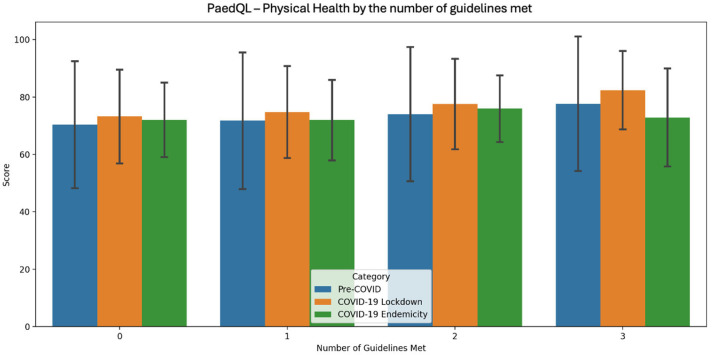
PaedQL score for physical health by the number of guidelines met across each time period of COVID-19.

Table 5, Figure 2, Figure 3 and Figure 4 presents the PedsQL scores for total health, psychosocial health, and physical health among pre-primary children in Singapore, categorised by the number of Singapore Integrated 24 h Activity Guidelines met across the three time periods.

During the pre-COVID period, a clear trend of improvement in all health metrics was evident as more guidelines were met, with the most pronounced improvement occurring when moving from two to three guidelines. In the COVID-19 lockdown period, all health metrics consistently improved as more guidelines were met, with physical health showing the most substantial improvement from 0 to 3 guidelines. During the COVID-19 endemicity period, the highest scores for all health metrics were observed when children met two guidelines, with psychosocial health showing the most significant improvement as adherence increased.

Table 6 presents the correlation of total health, psychosocial health, and physical health among pre-primary school children in Singapore and the number of Singapore Integrated 24 h Activity Guidelines met across the three time periods.

Across all periods, significant correlations were observed between the PaedQL metrics and various health outcomes. In the pre-COVID-19 period, psychosocial health and total health showed strong positive correlations (*p* < 0.001), with physical health also significantly correlated (*p* < 0.05). During the COVID-19 lockdown, both total health and physical health remained strongly correlated (*p* < 0.001), while psychosocial health showed a less strong correlation (*p* < 0.05). In the endemic period, total and psychosocial health continued to show significant positive correlations (*p* < 0.05), but the correlation for physical health was not statistically significant (*p* = 0.142). These findings suggest that psychosocial health and overall well-being maintained stronger correlations with health outcomes across the pandemic phases.

## 4. Discussion

The present study involved parent-reported information on preschool children who attended kindergarten in Singapore. The research objectives were to (a) explain the extent to which pre-primary children in Singapore adhere to the Singapore Integrated 24 h guidelines on PA, SL, and ST with comparison across three time periods of COVID-19: pre-COVID, during COVID-19 lockdown and during COVID-19 endemicity, and (b) how meeting the Singapore Integrated 24 h guidelines was in relation to children’s health-related QoL.

### 4.1. Physical Activity Guidelines

The Singapore Integrated 24 h Activity Guidelines recommends that preschool children should partake in a minimum of 180 min of total PA daily, with at least 60 min involving moderate-to-vigorous physical activity (MVPA) [48].

The number of children who met the PA guidelines significantly decreased from 32.7% to 27.4% during the COVID-19 lockdown (*p* < 0.01). Indoor and outdoor play significantly decreased during the COVID-19 lockdown (2.53 ± 1.58 h) compared to pre-COVID (2.76 ± 1.63 h, *p* < 0.001). Energetic play (MVPA) followed a similar pattern, decreasing during the lockdown (1.16 ± 1.10 h) compared to pre-COVID (1.28 ± 1.23 h, *p* < 0.01). This decline was anticipated due to the onset of the COVID-19 pandemic in 2020 and 2021, which brought lockdowns, restrictions on outdoor activities, playground closures, and limited access to sports facilities. Similar to Singapore, Japan and China’s urban density and cultural emphasis on academics likely contribute to lower PA adherence, exacerbated by stringent pandemic restrictions. In Japan, PA among preschoolers fell significantly, with weekday and weekend playtime decreasing by 8.5% and 15.3%, respectively, after the pandemic began. China also saw a decrease in adherence from 35.7% to 22.5% [37]. Sweden reported shifts in children’s movement behaviour, suggesting changes in adherence to activity guidelines [55]. However, some exceptions were also observed, such as in Alberta, Canada, it was found that preschoolers engaged in more overall and MVPA during childcare hours in the pandemic compared to before. It is likely that Canada, with an abundance of outdoor spaces, better supported PA during the disruptions [56].

Fortunately, our findings provide reassurance that, following the COVID-19 lockdown, the relaxation of restrictions and the return to indoor and outdoor activities have led to notable improvements in PA levels among pre-primary school children. The percentage increased to 34.4%, showing a recovery and a significant difference compared to the lockdown period. The total activity time (2.86 ± 1.64 h, *p* < 0.001) returned to pre-pandemic levels, and energetic play (1.37 ± 1.17 h, *p* = 0.002) has even exceeded the levels observed before COVID-19. Nonetheless, actions need to be taken to encourage healthier movement behaviours now that we are in the endemic period. 

### 4.2. Sleep Guidelines

The Singapore Integrated 24-Hour Activity Guidelines suggest that 5-year-olds should get 10–13 h of SL, while 6-year-olds should aim for 9–11 h [48].

Across the three time periods of COVID-19, SL duration was relatively the most consistent (Table 2), with a slight drop during the lockdown (from 9.09 ± 1.14 pre-COVID to 9.03 ± 1.26 h during COVID-19 lockdown) and a significant increase in COVID-19 endemicity to 9.21 ± 1.15 h (*p* = 0.0227).

In general, adherence to SL guidelines was the highest compared to the other 24 h activity guidelines. During the COVID-19 lockdown, adherence saw a modest but significant increase, rising from 33.4% prior to the pandemic to 37.9% during the lockdown. Fortunately, as restrictions were eased during the COVID-19 endemic phase, adherence not only maintained but also improved further to 40.6%, although this increase was not statistically significant (*p* = 0.3993).

The high adherence to SL guidelines is supported by another study in Singapore, which found that 70.2% of pre-primary children met the SL guidelines [57]. Additionally, several studies using the WHO guidelines have noted an increase in SL duration among preschoolers during the pandemic, indicating better adherence to SL guidelines. In Indonesia, it was also reported that an 8.0% increase in SL duration was observed during the COVID-19 pandemic [58]. Studies from European countries found that while children experienced an increase in total SL duration, their SL routines became irregular, which may have contributed to poorer SL quality [29,59]. Despite the disruption of children’s usual routines, without the need to wake up or go to bed early for school, children had greater flexibility in their SL schedule, which likely led to them sleeping and waking up later.

However, while there was a consistent and high adherence to SL guidelines, the actual duration of SL among Singaporean children typically only reaches the lower end of the recommended range, approximately 9 h per night. Although the guidelines were met, there is still potential for improvement in promoting greater optimal SL duration among pre-primary children in Singapore, regardless of the time period in this study.

### 4.3. Screen Time Guidelines

The Singapore Integrated 24-Hour Activity Guidelines recommend that pre-primary children limit their ST to less than 60 min per day [48].

However, adherence with these ST guidelines was found to be notably low across all three time periods, peaking at only 16.7% before the COVID-19 pandemic and falling significantly to a mere 10.8% during the COVID-19 lockdown (*p* < 0.001) (Table 3).

In terms of the usage duration of screens, pre-primary school children used significantly more ST during the COVID-19 lockdown, from 2.76 ± 2.31 h pre-COVID to 2.94 ± 2.48 h (*p* < 0.056).

This decline is expected, given the pandemic’s impact on daily routines. During the COVID-19 lockdowns from 2020 to 2022, increased ST was a natural consequence of home-based learning and restricted outdoor activities. The pandemic’s lockdown measures forced preschoolers to spend more time on screen-based devices like smartphones, tablets, and computers for both educational and entertainment purposes [27,28]. The closure of schools and childcare facilities, the lack of outdoor play and PA, and the shift to virtual learning further pushed children towards screen-based content for engagement [60]. Additionally, the adjustment to remote work for parents likely contributed to higher ST as a means of keeping children occupied [61].

Other research has similarly observed a decline in adherence to ST recommendations. For example, adherence among Japanese children fell from 27.2% before the pandemic to 19.9% during it [27]. In Australia, only 17.3% of children met the ST guidelines [62]. Additionally, in China, ST usage was found to have increased from 47.5% to 85.7% during the pandemic [37]. Shockingly, a study in Indonesia also found a dramatic 150.0% increase in average daily ST during the COVID-19 pandemic [38].

These findings point to global patterns of increased ST, though the scale of the increase varies widely by country. However, despite the different cultural attitudes toward technology, government policies, access to resources, and parenting norms, it appears that ST during the COVID-19 pandemic increased noticeably in most places.

In Singapore, although the percentage of ST guidelines met as outdoor activities resumed in the COVID-19 endemicity period remained low (10.6%), ST did still slightly decrease to 2.82 ± 2.40 h, though this change was not statistically significant compared to lockdown levels. An increasing number of young children are regularly interacting with both fixed and mobile screens. Therefore, it is essential to consider the potential long-term effects of ST and to tackle excessive usage at an early stage [63].

During the COVID-19 lockdown, there was a slight increase in the number of children failing to meet any guidelines (41.1%) compared to the pre-COVID (37.9%) and COVID-19 endemicity (36.5%) phases. The lowest proportion of children adhering to all three guidelines was also observed during the lockdown (0.7%), as opposed to pre-COVID (2.0%) and endemicity (2.1%), reflecting the adverse effects of restrictions on PA or changes in daily routines.

Thankfully, the endemicity period showed some recovery, with the percentage of children meeting two or all three guidelines improving to or exceeding pre-pandemic levels.

Notably, across all periods, 2.0% or fewer children managed to follow all three guidelines, underscoring the challenge of maintaining multiple health behaviours simultaneously regardless of whether there was an ongoing pandemic. However, it is also important to note that the majority of children in all periods met either none or only one of the guidelines, indicating that there is room for improvement across all time periods in helping children meet these health guidelines.

In terms of QoL, across all three time periods, health metrics consistently improved with greater adherence. The pre-COVID period showed a clear trend of improvement across all health metrics as more guidelines were met, with the largest boost seen when moving from two to three guidelines. During the COVID-19 lockdown, health metrics consistently improved with greater adherence, with physical health seeing the greatest gains from 0 to 3 guidelines. In the COVID-19 endemicity period, the highest health metric scores were observed when children met two guidelines, with psychosocial health showing the most notable improvement as adherence increased.

The positive correlations suggest that as more guidelines are met, QoL metrics tend to increase slightly. The consistency of these results across different time periods, including during the COVID-19 pandemic, also suggests a stable relationship between guideline adherence and QoL metrics.

Despite the analysis accounting for age and sex, which strengthens the validity of the findings by reducing potential confounding effects, the relationship is weak, suggesting that meeting more guidelines is associated with only slightly better QoL outcomes for children.

A possible explanation for the weak association might be due to the relatively small magnitude of changes in the guideline adherence measures over time. For example, while PA levels may have rebounded post-lockdown and guidelines have been met, the overall changes may not have been large enough to significantly influence the QoL metrics. Additionally, other moderating factors may have also played a larger role in determining these outcomes. The nature of the PaedQL used in this study integrates total health, including both physical and psychosocial health. The psychosocial component further breaks down into distinct domains such as school functioning, social functioning, and emotional functioning. Adherence to PA, ST, and SL guidelines may directly influence some aspects of QoL, such as physical health, but its impact on broader psychosocial domains is likely moderated by other environmental, social, and personal factors. These additional influences, such as family dynamics, socioeconomic status, and emotional support, may dilute the strength of the direct relationship between guideline adherence and QoL. These findings suggest that multiple factors, beyond guideline adherence alone, play a role in shaping children’s health and well-being. This interplay between different health behaviours and other influencing factors could help explain why there is only a weak correlation between adherence to the guidelines and QoL.

In conclusion, adherence to the 24 h movement guidelines, encompassing PA, ST, and SL recommendations, does still play a small role in the QoL and overall health outcomes of preschool children. Other research across different countries and settings has also consistently highlighted the positive associations between meeting these guidelines and various aspects of child well-being [53,64,65]. Understanding these associations is crucial for developing interventions and policies to promote the well-being and health outcomes of preschool children. By promoting and supporting adherence to these guidelines, caregivers, educators, and policymakers can contribute to the health of preschool-aged children.

### 4.4. The Importance of Establishing Healthy Lifestyle Behaviours in Pre-Primary Years

The present study reflects the state of lifestyle trends among pre-primary children aged 5 and 6 years old across three time periods of COVID-19, from 2018 to 2022. The overall trend shows that across all time periods, around four in five children are meeting none or only one of the recommended guidelines. Overall, children’s QoL scores tended to improve with the number of guidelines met. Despite the relatively weak strength of the associations, a statistically significant relationship exists between meeting more guidelines and achieving higher health scores.

These findings align with previous research, which shows that pre-primary children who adhere to more 24 h movement guidelines generally have better health-related QoL. For example, it was found that children meeting all three guidelines had significantly higher scores than those who did not [66]. This suggests that adequate PA, reduced sedentary behaviour, and sufficient SL positively impact children’s overall QoL. Additionally, following these guidelines has been associated with lower obesity rates and better mental health, both crucial for QoL [67,68].

The transition to formal schooling is a pivotal moment in a child’s life, where the establishment of good lifestyle behaviours can lead to enhanced school readiness skills, including both academic and social competencies [69]. Incorporating healthy lifestyle education can improve children’s cognitive and socio-emotional development, thereby facilitating a smoother transition into formal schooling [70]. This underscores the importance of integrating lifestyle education into early childhood curricula to promote long-term academic success and well-being.

Excessive ST, lack of PA, and insufficient SL are unfavourable lifestyle behaviours that contribute to an obesogenic environment rather than fostering an active and healthy lifestyle [71]. Recently, there has been a growing national focus on implementing additional safeguards and guidelines, particularly around limiting ST for young children, to address the mental and physical health risks associated with excessive and early exposure to digital media [72,73].

Research highlights that healthy lifestyle practices in early childhood offer protective benefits against non-communicable diseases and poor health outcomes later in life [74,75]. Therefore, establishing healthy lifestyle habits during the pre-primary years is vital, as these early behaviours set the stage for long-term habits that often persist into adulthood.

Parents, caregivers, and educators are key in shaping children’s lifestyle behaviours, as they are part of the immediate socio-ecological influences on the child [76]. During the pre-primary years, parents, caregivers, and teachers must make concerted efforts to prepare children for formal schooling, aiming for a smooth transition from preschool to primary school. Early intervention plays a key role in ensuring that children start building good lifestyle behaviours so as to start formal education with minimal difficulties.

#### 4.4.1. Implications

This study has presented findings that showcase important practical implications for multi-stakeholders, including parents, schools and policymakers, in promoting greater adoption of healthier lifestyles among children.

The rebound in PA following the lockdown is a positive sign, indicating that children were able to re-engage in physical activities once restrictions were lifted. This recovery suggests that the COVID-19 lockdown had a disruptive but temporary effect on children’s activity levels, and with the easing of restrictions, children could return to more active lifestyles. This rebound could also reflect the effectiveness of post-lockdown interventions, such as the reopening of outdoor spaces and recreational areas, which allowed children more opportunities to engage in PA.

However, the continued low adherence to ST guidelines post-lockdown raises significant concerns. Despite the easing of lockdown restrictions, screen time remained high, pointing to the potential long-term effects of lockdown habits, such as increased screen use during periods of home confinement. This persistent adherence gap suggests that efforts to reduce screen time may need to be more targeted and sustained, particularly as digital media continues to be integrated into daily routines both at home and in educational settings.

Therefore, policymakers, educators, and parents should continue to collaborate on promoting balanced routines that prioritise physical activity while limiting screen exposure.

The data underscore the need for stronger public advocacy by policymakers to focus on increasing physical activity (PA) and sleep (SL) while reducing screen time (ST) among children. The findings align with Singapore’s current direction, particularly in regulating screen time in educational settings. Additionally, Singapore has been expanding the development of outdoor and play spaces. However, more efforts are needed to actively encourage and support parents in taking their children outdoors to utilise these spaces effectively.

Children spend a significant portion of their weekdays in school, making it essential for schools to actively promote healthier lifestyle behaviours. Based on the findings from this study, schools should encourage more PA by incorporating movement into lessons whenever possible or by introducing regular breaks from sedentary activities and avoiding prolonged sitting. Furthermore, the successful adoption of healthier behaviours depends on strong collaboration between parents and schools. Fostering home-school partnerships that consistently reinforce healthy behaviours can help prevent confusion for children, ensuring alignment between the expectations set at school and at home.

The findings from this study highlight the importance of establishing a well-balanced routine that incorporates PA, ensures sufficient SL, and promotes less ST. Parents should lead by example, limiting their own screen time and encouraging more play activities as a family. This not only fosters familial bonding but also creates an environment conducive to physical activity while helping to reduce screen time for children.

#### 4.4.2. Strengths and Limitations

The participants in this study, regarding the ethnicity and gender of the preschool children, reflect the national demographic of Singapore. This study appears to be the first to compare the adherence to the newly launched Singapore Integrated 24 h Activity Guidelines over a five-year period and its correlation with health-related QoL.

However, a limitation of the study is that this was a cross-sectional study. While it can provide a valuable snapshot of the lifestyle behaviours of pre-primary children each year, the data do not allow for the establishment of causality as they do not track changes over the years [77,78]. Nonetheless, with the findings from this study, we are able to identify associations between adherence to guidelines and the health-related QoL of children each year. The number of valid responses collected during the COVID-19 endemicity period was smaller compared to the other two time periods. While the analysis was conducted using non-parametric tests that are more robust to unequal sample sizes, future studies should prioritise increasing participation and gathering more comprehensive data across the time periods. Additionally, the data are derived from self-reported online questionnaires, which may have been subjected to recall bias and social desirability bias. Parents who completed the questionnaires may have struggled to accurately recall or may have felt compelled to report more socially acceptable durations, potentially leading to inaccurate reporting of their children’s PA, ST and SL, resulting in overestimations or underestimations of these behaviours. Nonetheless, to minimise social desirability bias, the survey was conducted anonymously, and the questionnaires were carefully developed. Recall bias was also addressed by limiting the survey to a seven-day recall period.

#### 4.4.3. Future Studies

To mitigate recall bias and social desirability bias, future studies can use objective measures such as accelerometers to collect more accurate data and use them to cross-check with self-reported data. Future research should aim to assess the causal relationship between adherence to guidelines and developmental outcomes, as well as to examine the long-term effects of such adherence on children’s physical, socioemotional, and cognitive development. This will provide a deeper understanding of how following these guidelines impacts overall well-being and informs strategies to promote sustained positive outcomes.

## 5. Conclusions

Despite Singapore’s robust health measures and quick response to the pandemic, the COVID-19 pandemic and its different phases still have had varying impacts on preschool children’s adherence to ST, SL and PA guidelines. Children’s lifestyles changed across the different time periods, with the COVID-19 lockdown generally showing the most negative impact on play time and time engaged in MVPA, while the COVID-19 endemicity period showed some recovery and even improvements. This underscores the importance of continuing efforts to encourage adherence to guidelines as we transition into the post-pandemic era in Singapore.

While the associations between QoL and adherence to guidelines may be weak, it is still concerning that the number of children consistently meeting all three guidelines over the three time periods of COVID-19 remained alarmingly low, especially given the impact adherence can have on the QoL for pre-primary children.

Considering the major disruptions COVID-19 caused to children’s routines, it is essential to address the lingering effects on their lifestyle behaviours, with a focus on promoting recovery and fostering improvements. This requires the implementation of interventions that promote healthy habits, regular PA, and adequate SL.

However, addressing lifestyle behaviours in children is a complex and multifaceted challenge often referred to as a “wicked problem”. Achieving meaningful progress in adherence to guidelines demands sustained interventions that are not only evidence-based but also adaptable to evolving circumstances and contexts. Effective solutions require consistent monitoring of behavioural trends and outcomes, alongside the ability to remain flexible and responsive to changes. Given the intricate web of factors influencing lifestyle behaviours among children, a multi-stakeholder approach, with parents, schools, healthcare providers and policymakers working in unison, is necessary. A sustained, unified and comprehensive effort among key stakeholders is critical to empowering children to adopt and sustain healthy behaviours. By fostering a nurturing environment and prioritising collaborative efforts, we not only support children’s growth and development but also set the foundation for positive health outcomes [79].

## Figures and Tables

**Table 1:** Characteristics of participants across the time periods of COVID-19.

	Pre-COVID-19 (n = 1585)	COVID-19 Lockdown (n = 1209)	COVID-19 Endemicity (n = 340)
*N* (%)			
Gender			
Male	763 (48.2)	577 (47.7)	177 (52.1)
Female	811 (51.2)	625 (51.7)	163 (47.9)
Age			
5 years old	1116 (70.4)	758 (62.7)	243 (71.5)
6 years old	469 (29.6)	450 (37.2)	97 (28.5)
Ethnicity			
Chinese	971 (61.3)	715 (59.1)	221 (65.0)
Malay	264 (16.7)	239 (19.8)	44 (13.0)
Indian	214 (13.5)	158 (13.1)	46 (13.5)
Eurasian	4 (0.3)	3 (0.3)	1 (0.3)
Others	132 (8.3)	87 (7.2)	26 (7.7)

**Table 2:** Mean ± SD for PA, SL, and ST of child on an average day across the years.

	Pre-COVID-19	COVID-19 Lockdown	COVID-19 Endemicity
Physical activity (h)			
Indoor and outdoor play ***	2.76 ± 1.63 ^b^	2.53 ± 1.58 ^ac^	2.86 ± 1.64 ^b^
Energetic play **	1.28 ± 1.23 ^b^	1.16 ± 1.10 ^ac^	1.37 ± 1.17 ^b^
Sleep (h)			
Duration	9.09 ± 1.14	9.03 ± 1.26 ^c^	9.21 ± 1.15 ^b^
Screen Time (h)			
Duration	2.76 ± 2.31 ^b^	2.94 ± 2.48 ^a^	2.82 ± 2.40

Note: Energetic play is explained as physical activity that causes the child to “huff and puff” (i.e., moderate-to-vigorous intensity physical activity). ** denotes *p* < 0.01, *** denotes *p* < 0.001. ^a^ indicates a significant difference compared to the pre-COVID, ^b^ indicates a significant difference compared to the period during the pandemic, and ^c^ indicates a significant difference compared to the period after the pandemic.

**Table 3:** Percentage of children meeting PA, SL, and ST guidelines across the years.

	Pre-COVID-19		COVID-19 Lockdown		COVID-19 Endemicity	
	%	95% CI	%	95% CI	%	95% CI
Met physical activity guidelines **						
≥180 min of physical activity + ≥60 min of energetic play ^a^	32.7 ^b^	[30.3, 35.0]	27.4 ^ac^	[24.9, 29.9]	34.4 ^b^	[29.4, 39.5]
Met sleep guidelines **						
5 y/o: 10 to 13 h 6 y/o: 9 to 11 h	33.4 ^bc^	[31.1, 35.7]	37.9 ^ac^	[35.2, 40.6]	40.6 ^ab^	[35.4, 45.8]
Met screen time guidelines ***						
Screen media time < 60 min	16.7 ^bc^	[14.8, 18.5]	10.8 ^ac^	[9.0, 12.5]	10.6 ^ab^	[7.3, 13.9]

Note: Energetic play is explained as physical activity that causes the child to “huff and puff” (i.e., moderate-to-vigorous intensity physical activity). ** denotes *p* < 0.01, *** denotes *p* < 0.001. ^a^ indicates a significant difference compared to the pre-COVID, ^b^ indicates a significant difference compared to the period during the pandemic, and ^c^ indicates a significant difference compared to the period after the pandemic.

**Table 4:** Percentage of children who met none, one, two or all three guidelines each time period.

Number of Guidelines Met by Preschool Children	Pre-COVID-19		COVID-19 Lockdown		COVID-19 Endemicity	
	%	95% CI	%	95% CI	%	95% CI
0	37.9	[35.5, 40.2]	41.1	[38.3, 43.9]	36.5	[31.4, 41.6]
1	43.6	[41.2, 46.0]	42.4	[39.6, 45.2]	43.5	[38.3, 48.8]
2	16.5	[14.7, 18.4]	15.8	[13.7, 17.9]	17.9	[13.9, 22.0]
3	2.0	[1.3, 2.7]	0.7	[0.2, 1.1]	2.1	[0.5, 3.6]

**Table 5:** Mean ± SD of QoL scores for number of guidelines met.

		PedsQL Score		
		Total Health Score	Psychosocial Health Score	Physical Health Score
Period	No. of Guidelines Met	Mean ± SD		
Pre-COVID				
	0	69.99 ± 15.86	69.75 ± 15.05	70.35 ± 22.11
	1	71.79 ± 16.63	71.84 ± 15.18	71.72 ± 23.80
	2	73.57 ± 15.91	73.34 ± 14.29	74.02 ± 23.32
	3	77.47 ± 18.70	77.41 ± 17.54	77.65 ± 23.43
COVID-19 Lockdown				
	0	74.00 ± 14.42	76.21 ± 18.28	73.20 ± 16.31
	1	75.34 ± 14.56	77.00 ± 18.55	74.72 ± 16.02
	2	78.00 ± 14.18	80.12 ± 19.00	77.56 ± 15.72
	3	79.97 ± 10.34	80.44 ± 16.36	82.40 ± 13.67
COVID-19 Endemicity				
	0	73.70 ± 13.59	76.81 ± 19.70	71.98 ± 12.96
	1	73.21 ± 14.84	75.77 ± 21.08	71.93 ± 14.02
	2	79.37 ± 10.46	85.81 ± 12.28	75.92 ± 11.58
	3	76.40 ± 16.18	83.04 ± 19.67	72.86 ± 17.04

**Table 6:** Correlation between QoL Scores and Meeting Singapore Integrated 24 h Activity Guidelines Among Pre-Primary Children in Singapore.

Period	PaedQL Metric	ρ	*p*-Value	Effect Size (Cohen’s d)
Pre-COVID	Total health	0.092	<0.001	0.19
	Psychosocial health	0.102	<0.001	0.19
	Physical health	0.064	<0.05	0.14
COVID-19 Lockdown	Total health	0.095	<0.001	0.24
	Psychosocial health	0.067	<0.05	0.19
	Physical health	0.097	<0.001	0.24
COVID-19 Endemicity	Total health	0.116	<0.05	0.41
	Psychosocial health	0.135	<0.05	0.48
	Physical health	0.081	0.142	0.27

## Data Availability

The data associated with this study were published in the NIE data repository as per the requirements of the corresponding author’s university.
